# Using Dietary Indices—What’s Next?

**DOI:** 10.3390/nu12072161

**Published:** 2020-07-21

**Authors:** Sabine Rohrmann, Giulia Pestoni

**Affiliations:** Division of Chronic Disease Epidemiology, Epidemiology, Biostatistics and Prevention Institute, University of Zurich, Hirschengraben 84, 8001 Zurich, Switzerland; giulia.pestoni@uzh.ch

For centuries, it has been known that what we eat is essential for our health. Dietary advice has been an integral part of recommendations for a healthy life. However, for the majority of the world’s population, getting enough calories and a sufficient amount of nutrients (from amino acids to vitamins) has been a struggle, and overnutrition or a hypercaloric but “hyponutrient” diet is a rather new phenomenon for humans. Especially with respect to cardiovascular diseases (CVD), it has been shown that certain dietary behaviors, or dietary patterns, are associated with an increased risk of disease or even mortality [1]. One mechanism, by which a diet rich in calories, but lacking essential nutrients might act, is increased inflammation [2,3]. It has been postulated that chronic low-grade inflammation is associated with several types of CVD [4,5]. 

Dietary indices are frequently used to characterize certain aspects of diet, often its degree of adhering to certain dietary recommendations. The most well-known indices are probably the Healthy Eating Index [6] and the Alternate Healthy Eating Index [7], which aim to estimate the adherence to a healthy diet based on the consumption of vegetables, fruit, whole grains, sugar-sweetened beverages and fruit juices, nuts and legumes, red and processed meat, trans fat, long-chain n-3 fats, polyunsaturated fatty acids (PUFA), sodium, and alcohol. Both indices were associated with a reduced risk for chronic disease morbidity and mortality in different populations [8].

However, these well-known dietary indices were developed without considering the potential impact of dietary components on the mechanism through which diet influences the risk of diseases, such as inflammation. The Dietary Inflammatory Index (DII) assesses the inflammatory potential of different dietary components, based on their association with circulating levels of inflammatory markers [9,10]. Since its development in 2009 [9], the DII has been used in a variety of studies looking at a number of different health outcomes. Recently, Shivappa et al. conducted a systematic review and meta-analysis of studies on the association of DII with CVD incidence and mortality [11]. Fourteen studies, 11 cohort, two case-control and one cross-sectional, were deemed eligible for the analysis, which included 161,337 participants and 15,738 cases, covering a broad range of CVD outcomes. The main results were a positive association of a high DII (corresponding to a pro-inflammatory diet) vs. low DII with CVD incidence (relative risk (RR) 1.35, 95% confidence interval (CI) 1.11–1.63) and CVD mortality (RR 1.37, 95% CI 1.11–1.70). Looking more closely at CVD subgroups, there was an association between DII and the risk of myocardial infarction, but not stroke, angina pectoris, or ischemic heart disease. Some of these differences might be due to smaller numbers of studies that examined a particular outcome. For example, five datasets examined myocardial infarction as an outcome, but only three looked at ischemic heart disease. Interestingly, the results of CVD were similar in size by geographic region, but not by sex. A statistically significantly positive association was seen for women, but not for men; again, however, only few studies provided results stratified by sex.

The results of this meta-analysis provide further evidence that inflammation is likely one of the mechanisms by which an unhealthy diet might increase the risk of CVD. Other mechanisms, potentially interrelated with inflammation [12,13], are also likely to contribute to the development of CVD. For example, Tabung et al. developed insulin-related dietary indices [14] that capture the ability of whole diets to stimulate and/or sustain insulin secretion. On the other hand, certain types of dietary patterns, such as the Mediterranean Diet, the Nordic Diet or the Dietary Approaches to Stop Hypertension (DASH), have been shown to be linked with decreased inflammation [15,16,17] as well as improved insulin response and lipid profiles [18,19,20,21,22].

Whether inflammation, impaired glucose tolerance or blood lipids are the cause or the interlinked causes of chronic diseases is still a matter of debate [23,24], but, in either case, the question arises in which way diet is linked to them and how these associations can be transferred into effective dietary recommendations. How do we proceed with these interesting and important findings? Do we need a food pyramid with inflammation-reducing foods and in addition dietary guidelines that improve glucose tolerance and insulin sensitivity? Additionally, how can we make sure that those who are most in need of changing their diet towards a more healthy one do so?
